# Characterization of sulfated polysaccharide activity against virulent *Plasmodium falciparum* PHISTb/RLP1 protein

**DOI:** 10.12688/f1000research.26756.1

**Published:** 2020-10-23

**Authors:** Jennifer M. Mutisya, Victor A. Mobegi, Johnson K. Kinyua, Martha N. Kivecu, Raphael O. Okoth, Gladys C. Chemwor, Edwin W. Mwakio, Agnes C. Cheruiyot, Redempta A. Yeda, Charles O. Okello, Jackline A. Juma, Benjamin H. Opot, Dennis W. Juma, Amanda L. Roth, Hosea M. Akala, Ben M. Andagalu

**Affiliations:** 1 Department of Emerging and Infectious Diseases (DEID), United States Army Medical Research Directorate-Africa (USAMRD-A), Kenya Medical Research Institute (KEMRI)/Walter Reed Project, Kisumu, Kenya; 2 Department of Biochemistry, Jomo Kenyatta University of Agriculture and Technology, Nairobi, Kenya; 3 Department of Biochemistry, University of Nairobi, Nairobi, Kenya

**Keywords:** Exported proteins, Sulfated polysaccharides, PHISTb/RLP1, Interactions, P. falciparum, Anti-malarials

## Abstract

**Background: **The emergence of artemisinin resistance in South East Asia calls for urgent discovery of new drug compounds that have antiplasmodial activity. Unlike the classical compound screening drug discovery methods, the rational approach involving targeted drug discovery is less cumbersome and therefore key for innovation of new antiplasmodial compounds. *Plasmodium falciparum* (Pf) utilizes the process of host erythrocyte remodeling using Plasmodium-helical interspersed sub-telomeric domain (PHIST) containing proteins, which are amenable drug targets. The aim of this study is to identify inhibitors of PHIST from sulfated polysaccharides as new antimalarials.

**Methods: **251 samples from an ongoing study of epidemiology of malaria and drug resistance sensitivity patterns in Kenya were sequenced for PHISTb/RLP1 gene using Sanger sequencing. The sequenced reads were mapped to the reference Pf3D7 protein sequence of PHISTb/RLP1 using CLC Main Workbench. Homology modeling of both reference and mutant protein structures was achieved using the LOMETs tool. The models were refined using ModRefiner for energy minimization. Ramachandran plot was generated by ProCheck to assess the conformation of amino acids in the protein model. Protein binding sites predictions were assessed using FT SITE software. We searched for prospective antimalarials from PubChem. Docking experiments were achieved using AutoDock Vina and analysis results visualized in PyMOL.

**Results: **Sanger sequencing generated 86 complete sequences. Upon mapping of the sequences to the reference, 12 non-synonymous single nucleotide polymorphisms were considered for mutant protein structure analysis. Eleven drug compounds with antiplasmodial activity were identified. Both modelled PHISTb/RLP1 reference and mutant structures had a Ramachandran score of >90% of the amino acids in the favored region. Ten of the drug compounds interacted with amino acid residues in PHISTb and RESA domains, showing potential activity against these proteins.

**Conclusion: **These interactions provide lead compounds for new anti-malarial molecules. Further *in vivo* testing is recommended.

## Introduction

African countries have 94% of malaria cases and the highest malaria-related death rates according to the 2019 World Health Organization (WHO) Malaria Report ( WHO, 2019). Despite its prioritization in the Millennium Development Goals and other large scale global health initiatives, efforts and strategies to reduce the burden of malaria by 40% have stalled over the years due to different challenges ( WHO, 2018). These challenges include emergence of *Plasmodium falciparum* resistance to first-line treatment, the lack of an efficacious vaccine, vector resistance to insecticides, the great diversity of malaria parasite, and insufficient funding towards the control of the disease. These challenges have created a gap that calls for quick intervention to reduce the burden of the disease ( Dondorp *et al*., 2012). The latest drug resistance development of *P. falciparum* to artemisinin combination therapies in Southeast Asia ( Takala-harrison *et al*., 2015), calls for the development of new drug compounds that can overcome parasite resistance to existing drugs.

Studies have shown that sulfated polysaccharides have antimalarial activities ( Mourão, 2015). Unlike most drugs that target the blood stages of the parasite, these compounds exploit newly identified pathways to interact with intracellular parasites. It has been established that merozoites perforate the erythrocyte membrane before egress ( Boyle *et al.*, 2010). Heparin enters the infected erythrocyte through these pores and prevents merozoite egress ( Glushakova *et al*., 2017). These findings have led to therapies using heparin to treat severe malaria ( Vogt *et al*., 2006). Moreover, heparin has been shown to reduce the cytoadherence of infected red blood cells and thus reduce parasitemia. However, heparin use was discontinued due to the induction of severe bleeding in patients ( Leitgeb *et al*., 2011). Low concentrations of heparin were later used successfully in safety and efficacy trials to disrupt cytoadherence and rosette formation and additional research has been conducted to identify low anticoagulant sulfated polysaccharides with antimalarial activity. Modified heparin compounds and polysaccharide inhibitors were successfully profiled for activity against intracellular parasites ( Boyle *et al*., 2017). These compounds have been identified in marine organisms and plants ( Marques *et al*., 2016).

The interaction of sulfated polysaccharide molecules with merozoite proteins and the *P. falciparum* erythrocyte membrane protein family have been studied intensively. Members of the Duffy binding-like domain and reticulocyte binding-like domain were shown to have interaction with heparin at different affinities ( Saiwaew *et al*., 2017). In addition, sulfated polysaccharides interacts with all *P. falciparum* reticulocyte binding homologues, including PfRH2, PfRH4 and PfRH5 ( Somner *et al*., 2000). The *P. falciparum* parasite uses not only the merozoite surface proteins for invasion but also exported proteins that are found on the surface of the erythrocyte to form cross-linkers with erythrocytes ( Tarr *et al*., 2014). The exported proteins have been classified to be essential for parasite survival and virulence, hence contribute to the pathogenesis of malaria ( Maier *et al*., 2008). The Plasmodium-helical interspersed sub-telomeric domain (PHIST) family of exported proteins are expressed in most *Plasmodium* species but they are largely expanded in *P. falciparum*. The PHIST domain clusters into three subgroups across the *Plasmodium* genus, PHISTa, PHISTb, and PHISTc. PHISTa and PHISTb localize specifically in *P. falciparum*. PHISTb is found in *P. vivax* and *P. knowlesi.* One major virulent protein of the PHIST family is PHISTb containing ring-infected erythrocyte surface antigen (RESA)-like protein, abbreviated to PHISTb/RLP1 (PlasmoDB accession number, PF3D7_0201600). This protein has been involved in enabling attachment of infected erythrocytes to organs and blood vessels, as well as erythrocyte remodeling mechanism. Studies have associated this protein with controlling expression mechanism of PfEMP1 ( *P. falciparum* erythrocyte membrane protein) ( Moreira *et al*., 2016; Oberli *et al*., 2016; Warncke *et al*., 2016). As a drug target in the *P. falciparum* genome, PHISTb/RLP1 is identified in Tropical Disease Research Targets database (TDR Targets ID: 3288)

In this study, we profile specific protein-ligand interactions that give the first insight into drug compounds targeting exported proteins. Furthermore, we outline old and novel mutations found within PHISTb/protein sequences from whole blood samples collected from various malaria endemic sites in Kenya. We link the effects of these mutations on protein structure, active sites and interaction with the drug molecules.

## Methods

### Study setting

The study was conducted at the Malaria Drug Resistance laboratory in Kisumu, Kenya, under the United States Army Medical Research Directorate-Africa and Kenya Medical Research Institute. The study analyzed a subset of archived *P. falciparum* samples from ongoing approved research, studying the epidemiology of malaria drug resistance patterns in Kenya. Samples were collected from the following sites: Kisumu and Kombewa (endemic regions), Kericho and Kisii (highland epidemic areas), Marigat (seasonal transmission zone), and Malindi (coastal area with declining endemicity). The proportion of samples collected was roughly the same between the six collection sites

### Sample collection

Samples were collected from volunteers consenting to participate in the study, who were aged 6 months. 2.5 ml of venous blood was collected from participants presenting signs of uncomplicated Malaria. 0.5ml of the blood was transferred to the Acid Citrate Dextrose tubes for sterile culture. At least 1ml of the blood was transferred into an ethylene glycol-bis(2-aminoethylether)-N,N,N′,N′tetraaceticacid (EDTA) tube for molecular assays which included DNA extraction. From the remaining blood sample, 3–5 drops of the syringe blood sample was transferred onto Whatman filter paper by blotting on three separate spots and archived at room temperature in a pouch.

### Ethical clearance

Written informed consent was provided by participants and/or their legal guardians. The study was carried out in accordance to approved guidelines by the Ethical Review Committee of the Kenya Medical Research Institute (KEMRI), Nairobi, Kenya and Walter Reed Army Institute of Research (WRAIR) Institutional Review Board, Silver Spring, MD. The study was conducted under the approved study protocols KEMRI #1330/WRAIR #1384 and KEMRI #3628/WRAIR #2454.

### Sequencing of PHISTb/RLP1 gene using molecular assays

A total of 251 viable samples were used in this study. *P. falciparum* parasite DNA extraction was carried out using Qiagen DNA Mini extraction spin protocol (Qiagen, Valencia, CA) as per manufacturer’s instructions. Extracted DNA was stored at -20 ^°^C for preservation.

### *Plasmodium* testing and *P. falciparum* speciation using quantitative PCR

First the samples were tested for the presence of *Plasmodium* parasite *. Plasmodium* genus was tested with primers F1 and R1 shown in Table 1, the probe was labeled with FAM (6-carboxyfluorescein) in 5’ and at the 3’ TAMRA (6-carboxytetramethyl-rhodamine). All the *Plasmodium* positive samples were then tested for *P. falciparum* species. *P. falciparum* species-specific assay was carried out with primers and probes designed with FAM reporter, as reported in previous work ( Kamau *et al*., 2013; Veron *et al*., 2009). The samples were amplified in 0.1 milliliters 96-well plates. The kit used for this assay was Quantifast (QIAGEN). The components of each well were, 2µl DNA template, recommended amount of Quantifast master mix and recommended amount of primers and probes. The reaction mixture amounts were calculated according to the number of samples run per assay.

**Table 1.** Primers and probes used to diagnose *Plasmodium* parasite and detect *Plasmodium falciparum* species in real time PCR. These are primers and probes were published in previous work by Kamau *et al.* (2013).

	Primer/probe name	Primer sequence
***Plasmodium primers***	F1	5’-GCTCTTTCTTGATTTCTTGGATG-3’
	R1	5’-AGCAGGTTAAGATCTCGTTCG-3’
	Probe (FAM-TAMRA)	5’-ATGGCCGTTTTTAGTTCGTG-3’,
***Plasmodium falciparum***	*Pf*-1	5’ATTGCTTTTGAGAGGTTTTGTTACTTT3’
	*Pf*-2	5’GCTGTAGTATTCAAACACAATGAACTCAA3’
	Probe (FAM-MGB)	5’CATAACAGACGGGTAGTCAT3’

The real-time PCR assay was the carried out in Applied Biosystem Quantstudio 6 Flex (Quantstudio ^Tm^ real-time PCR applied Biosystems by Thermos Fisher Scientific) with the following conditions: 95°C for 10 minutes, 40 cycles at 95°C for 15 minutes, and then 60°C for 1 minute, as previously published ( Kamau *et al*., 2013). RNAse-P was used the housekeeping gene and Ct value was awarded by the equipment as the value for which the amplification was high enough to pass the detection threshold The results were exported into Excel worksheet showing the cycle threshold (CT) value for each sample. Only samples positive by *P. falciparum* confirmatory PCR with a cycle threshold value of at least 32 and below were considered for downstream assays ( Table 1).

### Conventional PCR assays for *P. falciparum* PHISTb/RLP1 gene amplification and gel electrophoresis

Gene-specific primers were designed using Primer3Plus, as described in the literature ( Untergasser *et al*., 2012). Mapping of the primers to sequences to test the correct annealing on sequence was achieved using Sequence Manipulation Suite version 2. Primers are included in Table 2.

**Table 2.** PHISTb/RLP1 specific gene primers for *Plasmodium falciparum*, as designed in Primer3 plus tool. The table shows the regions on the gene where each primer should anneal as well as the expected PCR product. These are novel primers for this gene, designed and used in this study for the first time.

Gene	Region	Primers Forward & Reverse	PCR Product
*Pf*PHISTb/RLP1	80-998	>RLP1_F1: 5’-CAATGACCATGTGTTCAGA-3’	919
		>RLP1_R1: 5’-TCTGGAGATAAAGCATACCC-3’	
*Pf*PHISTb/RLP1	791-1450	>RLP1_F2: 5’-GCAAAACCAACGTGTAATC-3’	660
		>RLP1_R2: 5’-TTCAACATCTCTTTCGACTG-3’	

PCR was carried out in Applied Biosystems thermocycler machine, the reaction conditions were as follows: the first pair of primers amplifying the first fragment were 94°C for 5 minutes during initial denaturation phase and 30 seconds in the normal denaturation, annealing 59°C for 30 seconds, and extension 72°C for 1 minute and 5 minutes in the final elongation stage. The second primer pair annealing temperature was 58°C for 30 seconds while the denaturation and extension phases were the same as primer pair one. We used the puRE Taq Ready-To-Go PCR beads standard master mix (GE Healthcare, CITY, STATE), as per manufacturer’s instructions.

Following successful amplification, PCR amplicons were visualized in 2% agarose gel electrophoresis for confirmation. The 2% agarose gel was prepared using 2.25gm of agarose powder in 150 ml of 10% Tris Acetate EDTA (TAE) buffer. The gel was then heated in the microwave for 3 min to allow mixing. The gel was then cooled until it was lukewarm, then 15µl of gel red was added. Thereafter the gel was poured into the gel tank in which gel combs had been inserted. The gel was incubated at room temperature for 30 to 60 minutes to allow the gel to set. Upon setting, the gel was flooded with TAE running buffer just above the wells for loading of samples. Samples were then loaded into the wells on the gel wells by mixing 4µl of the sample with 4µl of loading dye. Additionally, 1.5µl each of 1kb hyper ladder 1 (Bioline) was loaded into the first and final wells on the gel. After loading all the samples, the gel was completely flooded with running buffer (10% TAE) and was run at 230 voltage for 50 min. The gel was then read on UVI save HD5 - Gel documentation (Uvitec Limited, United Kingdom).

### Sanger sequencing of *P. falciparum* PHISTb/RLP1 gene

Amplification products were purified using ExoSAP-IT (Affymetrix, CITY, STATE) enzymatic cleanup procedure. The enzymatic reaction involves an exonuclease enzyme Shrimp Alkaline Phosphatase (SAP) enzyme, which cleaves phosphate group from unincorporated dNTPs. Using 0.2 ml 96 well plates, each well contained 2µl of ExoSAP-IT enzyme mixed with 8 µl of PCR product. The plates were incubated at 37°C for 20 minutes to allow the enzymes to work then for a further 20 minutes at 80°C to inactivate the ExoSAP-IT enzymes prior to sequencing. Big dye termination PCR was carried out using primary PCR primers and amplification conditions. Big dye terminator sequencing is a modification of Sanger sequencing where dideoxynucleotides (ddNTPs) labeled with a specific fluorescent dye corresponding to each nucleotide base are added to the reaction. The sequencing products were cleaned using Sephadex supplied by Sigma. 10 µl of HiDi formamide (Applied Biosystems) was added into the purified sequence products. The plates were then sealed and heated at 96°C for 3 minutes to denature the DNA and then analyzed using capillary electrophoresis ABI 3130/3500xL Genetic analyzer. Read assembling was done on Qiagen CLC main Workbench version 8.0.1 ( CLC Bio, 2014).

### Single nucleotide polymorphisms analysis in PHISTb/RLP1 protein sequences

Multiple sequence alignment was achieved using Multiple Sequence Comparison by Log-Expectation ( MUSCLE) ( Edgar, 2004) in Jalview version 2.11.0 ( Alzohairy, 2014; Waterhouse *et al*., 2009). Bioedit version 7.2.5 ( Hall, 1999) was used to enable intron and gap deletions, as well as translation of the nucleotides to protein sequences. The alignment was copied to Microsoft Excel and frequencies of the observed single nucleotide polymorphisms (SNPs) counted, as well as generation of frequency bar graphs.‬‬‬‬‬‬‬‬‬‬‬‬‬‬‬‬‬‬‬‬‬‬‬‬‬‬‬‬‬‬‬‬‬‬‬‬‬‬‬‬‬‬‬‬‬‬‬‬‬‬‬‬‬‬‬‬‬‬

### Protein 3D structure modelling, model verification and binding site predictions

Protein structures of the PHIST/RLP1 reference (Pf3D7) and mutant proteins structure were predicted via *ab initio* multithreading tool LOMETS ( Wu & Zhang, 2007; Zheng *et al*., 2019). The LOMETS tool has an internal selection of templates through in-built multiple sequence alignment and ranking the templates in descending order according to a normalized Z score. A Z score greater than or equal to one is considered a good alignment.

Protein Data Bank (PDB) hits used as templates for the PHISTb/RLP1 reference structure were 4jle, 5ez3 and 6d03 all with a normalized Z-score ≥1. The templates selected for the PHISTb/RLP1 mutant structure modelling were 4jle, 2ziq and 6d03, which had a normalized z-score ≥ 1. Two templates were similar for the reference and mutant protein structure threading because of sequence similarities at the starting and ending domains of the the protein sequences. The middle template was different for both protein structure threading of mutant and reference structures due to presence of point mutations in this region of the mutant PHISTb/RLP1 protein sequence.

The models were submitted to ModRefiner ( Xu & Zhang, 2011) for energy minimization. The energy minimized structure of PHISTb/RLP1 reference had a Template Model (TM) score of 0.97 to the initial model. The TM score is calculated between 0 and 1, whereby the higher the TM score the higher the perfect match between the two structures. The models were verified using Galaxy Refine web server tool ( Ko *et al*., 2012) for correction of wrong rotamers. The protein model stability were validated through the parameter of percentage residues lying within the favored and allowed regions using Rampage tool ( Lovell *et al*., 2003) and overall stability confirmed using PROCHECK web tool ( Laskowski *et al*., 1993). Following model validations, the functional sites within the protein structure were predicted in the webserver tool FT Site ( Ngan *et al.,* 2012)( Brenke *et al*., 2009). FT SITE algorithm was reported to achieve near experimental accuracy of predicting druggable hotspots in 94% of apo-proteins used in evaluation of binding sites methods. For confirmation of our binding site clusters, COACH, a metaserver tool that uses comparisons of other servers prediction to profile highly accurate protein-ligand binding sites, was used ( Yang *et al*., 2013).

### Sulfated polysaccharides search and toxicity analysis

The sulfated polysaccharides containing anti-malarial properties were identified through chemical modifications, as per previous research ( Boyle *et al*., 2017). We searched for these compounds from the PubChem database, which stores chemical structures of identified chemical compounds and their biochemical activities ( Kim *et al.,* 2016). The drug likeness of the screened compounds were analyzed for the Lipinski Rule of Five, as follows: molecular mass <500 Dalton; high lipophilicity (expressed as LogP <5); <5 hydrogen bond donors; <10 hydrogen bond acceptors; and molar refractivity should be between 40–130. This was achieved using the Lipinski Rule of Five webserver ( Gimenez *et al*., 2010)

### Preparation of proteins and ligands, molecular docking and output visualization

The Standard Database Format (.SDF) files of the drug compounds obtained were converted to PDB format files using Open Babel tool version 2.3.1 on Linux command line ( O’Boyle *et al*., 2011). Ligand files, receptor files (protein models), and grid parameters were prepared using MGL tools version 1.5.6 ( Morris *et al*., 2009). Proteins (receptors) and the ligands (drug compounds) were converted from PDB format to PDBQT file format required by autodock tolls. Docking simulations were achieved using AutoDock Vina version 1.1.2 ( Trott & Olson, 2010). The output of AutoDock Vina results was visualized using PyMOL version 2.3.5 (Schrodinger LLC).

## Results

### Diagnosis for *Plasmodium* genus and *Plasmodium falciparum* species detection

A total of 175 out of 251 (70%) samples tested positive for *Plasmodium* parasite. The 70% positive samples were further tested for *P. falciparum* species and 63% were positive. The positive samples (n=110) were used for the downstream experiments, i.e. they were used to sequence PHISTb/RLP1 gene.

### Genetic mutations in PHISTb/RLP1 sequences

102 of the 110 samples processed for Sanger sequencing yielded sequences. Of these, 86 samples generated complete sequences; 16 samples had poor sequences.

### Non-synonymous SNPs identified in the PHISTb/RLP1 sequenced data

A total of 157 non-synonymous SNPs were observed across the full length of the protein sequences (485 amino acid in length). The SNPs occurred at different frequencies in the total sequenced samples. Only SNPs occurring at a frequency >50% were considered for protein structure analysis (n=20) ( Table 3).

**Table 3.** Total observed non-synonymous SNPs in the sequenced data after read cleaning, multiple sequence alignment, intron deletion and conversion to protein sequences. The total number of SNPs was 157 across the entire protein length, in all the 102 sequenced samples. 107 SNPs were only overserved in 1-10% of the samples and were excluded. 30 of the remaining SNPs occurred in 10-50% samples and this frequency was not high enough to be considered for downstream analysis, they were excluded as well. The final 20 codons of the total observed non-synonymous SNPS had a frequency >50% of the total samples sequenced. These were considered for protein structure analysis. The frequency was high thus the implications of these SNPs was analyzed further.

Non-synonymous SNPs (n=157)	Frequency in 102 samples
107	10% and below
30	between 10% to 50%
20	Above 50%

### SNPs considered for mutant protein structure analysis

**Figure 1.**
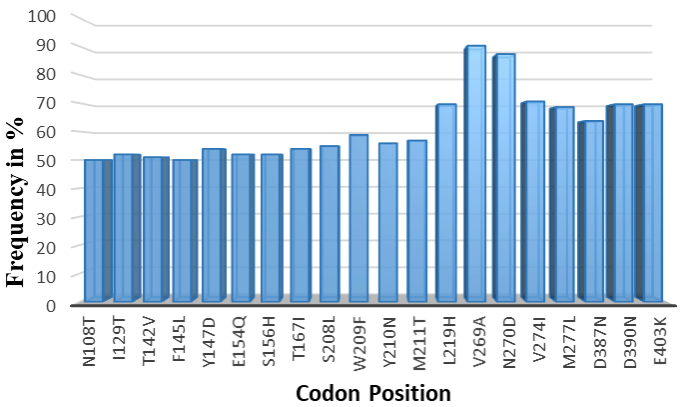
High frequency non-synonymous SNPs observed in sequenced data for PHISTb/RLP1 protein. Non-synonymous SNPs occurring in 50% and above of the total sample size. Codons I129T, T142V, Y147D, E154Q, S156H, T167I, S208L, M211T, L219H, D387N, D390N, and E403K were substitution of amino acids across different functional groups. These were modelled and their implications on protein structure and interactions with a drug compound investigated.

Within the 20 SNPs occurring at high frequency, codons F145L, D146R, Y147D, S156H, S208L and L219H were all novel mutations identified in our samples across all different sites where our samples were collected. We compared our data against SNPs recorded in PlasmoDB genetic variation tracks for the PHISTb/RLP1 3D7 reference ( PF3D7_0201600). These six mutations were not present in the database; therefore, they could be newly identified mutations for PHISTb/RLP1 gene as observed in our samples. Analyzing the amino acids substitutions further, we observed that mutations at codons N108T, F145L, W209F, Y210N, V269A, V274I and M277L were all substitutions of amino acids within the same group. The F145L, W209F, V269A, V274I and M277L mutations were all substitutions within the non-polar group, while N108T and W209F were substitutions in the polar group. Amino acids within the same group play similar functional roles in the protein sequence and thus these SNPs were not considered for protein function analysis. On the other hand, I129T, T142V, Y147D, E154Q, S156H, T167I, S208L, M211T, L219H, D387N, D390N, and E403K were substitutions of amino acids across different functional groups. We postulated that these point mutations are most likely to change the folding of the protein structure and have an effect on the function of the protein. These codons were modelled in the mutant protein sequence for PHISTb/RLP1 structure. Their effect on the tertiary protein structure as well as effect on interaction with a drug compound were further investigated ( Figure 1). 

### Homology modeling, structure refinement and binding sites prediction

PHISTb/RLP1 reference and mutant structures were modelled successfully. The LOMETs tool gave five models for the reference and mutant structure each. The fourth model in both reference and mutant modelling sessions was selected and submitted to ModRefiner for energy minimization. The energy minimized structure of PHISTb/RLP1 reference had a TM score of 0.97 to the initial model. The TM score is calculated between 0 and 1, whereby the higher the TM score the higher the perfect match between the two structures **.**The PHISTb/RLP1 mutant protein had a TM score of 0.98 following energy minimization. The models were submitted to Galaxy Refine for further model refinement and then we chose the first models with best scores. The Galaxy refined models were submitted to RAMPAGE software for Ramachandran plot assessment. The reference structure had 95% and the mutant structure 94% of the residues in the favored region. The PROCHECK results displayed 92% of the residues in the reference structure to be in the most favored region. The mutant structure had 90% of the residues to be in the favored region. Ramachandran plot analysis required a good model to have at least 90% of the amino acids in the favored region. These Ramachandran plot scores qualified the two models to be used for the docking experiments and were submitted to binding sites prediction tools.

**Figure 2.**
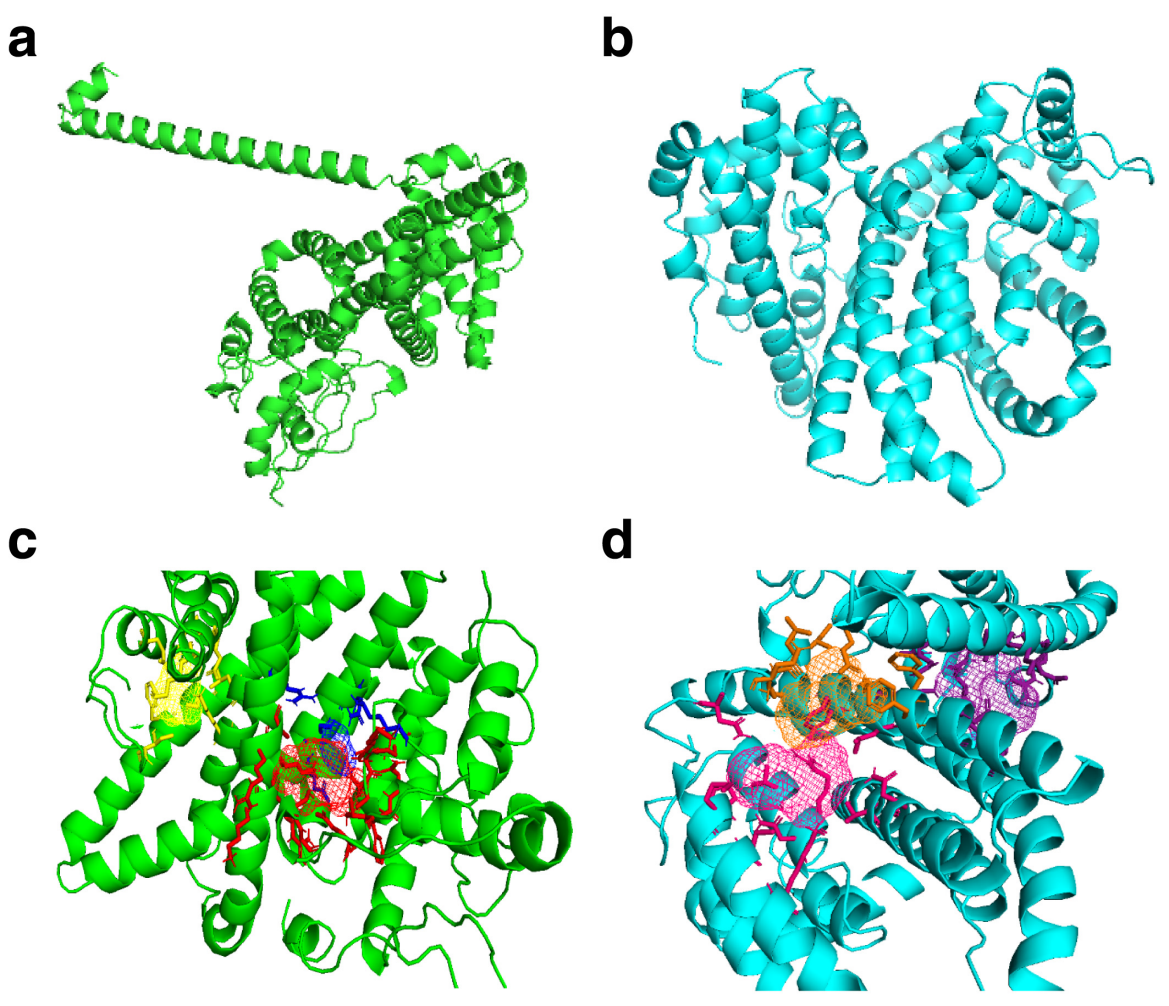
PHISTb/RLP1 reference and mutant homology modelled structures and their predicted binding sites clusters. ( **a**) PHISTb/RLP1 reference protein structure shown in green; ( **b**) structure of PHISTb/RLP1 mutant protein structure shown in cyan; ( **c**) PHISTb/RLP1 reference protein binding site clusters, site 1 in red, site 2 in blue and site 3 in yellow; ( **d**) PHISTb/RLP1 mutant protein binding sites clusters, site 1 in pink, site 2 in orange and site 3 in dark purple. All models visualized in PyMOL.

FT Site prediction outcome included Photoshop Element (.pse) files that were visualized in PyMol to visualize the active amino acids in the binding sites clusters for both reference and mutant PHISTb/RLP1 protein structures, as shown in Table 4. We also considered residues that were predicted by COACH software and had the highest confidence score (C-score) of 0.05 for PHISTb/RLP1 reference protein structure. COACH predicted residues in PHISTb/RLP1 protein structure had a C-score between 0.07 and 0.08. The C-score ranges between 0–1 where a higher C-score gives a more reliable prediction. In both the mutant and the reference structures, there were active residues predicted by both tools. This double prediction emphasized the accuracy of the binding sites as functional regions of the proteins ( Figure 2 and Table 5).

**Table 4.** Galaxy refine scores for the best model of the protein structures for PHISTb/RLP1 reference and mutant proteins after structure refinement. The score considered included the poor rotamers scores in which they are very low in both inferring to correction in R chain conformations. The Rama favored scores were also used to consider the models. The scores above 90 for Rama favored shows that the models are good to be used for further analysis.

Model	GDT-HA	RMSD	MolProbity	Clash Score	Poor Rotamers	Rama Favored
PHISTb/RLP1 Reference	0.9454	0.433	2.346	22.9	1.1	94.8
PHISTb/RLP1 Mutant	0.9438	0.436	2.348	25.7	1.5	93.6

**Table 5.** Amino-acids found within binding sites clusters in both reference and mutant PHISTb/RLP1 protein structures as predicted by FT-Site and COACH softwares. The highlighted residues are predicted by both softwares. This puts emphasis on the accuracy of the predictions.

Protein Structure	FT-Site Binding Predictions	COACH Meta-server redictions	Residues Predicted in Both Tools
PHISTb/RLP1 Reference	K80, K84, K131, S132, A133, F134, N135, S161, E165, G162, T167, F241, S245, K205, K314, R318, A319, K128, N197, S201, E204, K205, R280, L282, K285, K376, E379, D380, E403 and I412	F83, T86, F87, N90, G162, I194, N197, K200, A319, E422 and Y423	N80, F241, K205 and E403
PHISTb/RLP1 Mutant	I15, L16, D17, N18, N20, P27, M28, C31, K35, T86, R89, N90, T211, N214, L244, Y248, F241, F242, S245, N233, K246, T289, E292, E293, I412, V413, G414, A415, N416, E422, D448, V449, E452 and R456	N210, L244, K247, K285, T286, F102, K107, V198, S201, E204, K205, L244, Y328 and E329	K35, R89, N90, L244 and Y248

### Toxicity analysis of sulfated polysaccharides

The PubChem database search yielded the following compounds: beta carrageenan, alpha carrageenan, dextrin sulfate, amylopectin sulfate, zinc sulfate, ghatti sulfate, 2,4-diaminoanisole sulfate, cyclodextrin sulfate, Fucoidan, 3-aminophenylboronic acid and 3,6-Di-O-benzoyl-D-galactal. In this group of compounds, dextrin sulfate deviated from the Lipinski Rule of Five. Beta carrageenan has hydrogen bond acceptors of 12, the compound was considered for interaction experiments because the Rule of Five allows a window of one deviation of the physical properties. 3-aminophenylboronic acid PDB file contained atoms that could not be recognized by AutoDock Vina. The compound was left out for docking experiments. All the other drug compounds adhered to Lipinski Rule of Five hence passing the toxicity test of drug likeness and were tested for activity against the exported protein PHISTb/RLP1 ( Table 6).

**Table 6.** Screened sulfated polysaccharides searched from PubChem database. Accession numbers, physical properties and Lipinski Rule of Five properties of each compound are curated. The Lipinski rule of five requires that a drug compound meets all the properties or deviates with at most one property. All the screened compound met the rules of a drug compound, except dextrine sulfate.

Compound	CID Number	Molecular weight g/mol	Hydrogen bond donor count	Hydrogen bond acceptor count	High lipophilicity (Log P)	Molar Refractivity
Alpha carrageenan	102199625	416.394	4	12	1.7	85
Beta carrageenan	102199626	336.337	4	9	1.9	75
Dextrin sulfate	129722329	598.478	11	20	1.2	113
Amylopectin sulfate	23675774	190.189	0	4	0.8	40
Zinc sulfate	24424	161.436	0	4	0.2	15
Ghatti sulfate	3423265	332.431	0	5	3	78
2,4-Diaminoanisole sulfate	38221	236.242	4	7	-0.1	49
Cyclodextrin sulfate	71317197	322.304	0	8	1.7	68
Fucoidan	92023653	242.242	3	7	1	50
3-Aminophenylboronic acid	92269	136.945	3	3	1	38
3,6-Di-O-benzoyl-D-galactal	11348785	354.348	1	6	3	83

### PHISTb/RLP1 protein-ligand interactions

The identified compounds interacted with the target protein. Alpha carrageenan, amylopectin sulfate, cyclodextrin sulfate and Fucoidan exhibited optimum interactions with the PHISTb/RLP1 protein. The amino acids interacting with these compounds were identified in the binding sites. Of these interactions, amino acids S132, K84 and N80 were found within the PHISTb domain. These interactions significantly show the potential of interfering with the function of the exported protein. Beta carrageenan and dextrin sulfate compounds had specific interactions with the target protein. Amino acids K376, E402 and E403 were identified in the binding site clusters. 2, 4-Diaminoanisole sulfate and zinc sulfate interactions with the protein were not specific with the identified binding site clusters. However, we noted that the amino acids interacting with these compounds are within the PHIST domain which spans amino acids 1 to 167 of the protein sequence. Ghatti sulfate showed interaction with the reference protein as well, although not specific.

The same drug compounds were tested on the m odelled PHISTb/RLP1 mutant protein structure with the identified non-synonymous SNPs from the sequenced data. The aim was to test whether mutations found within the protein interfere with the interactions or not. The interactions of the drug compounds with mutant protein were not as strong as those of the reference protein. The interactions in the mutant protein occurred at different binding residues because the mutations within the proteins affected the folding of the protein structure. Due to these mutations, there was a shift of the binding sites clusters. Alpha carrageenan, ghatti sulfate and 3,6-Di-O-benzoyl-D-galactal depicted specific interactions with the mutant protein. Residues K35, N90, D17 and N18 were within the PHIST domain. The other compounds were interacting with different residues in the protein domains. The interactions were not optimized to the predicted binding site clusters. However, we noted that some of the interacting residues were within the PHISTb domain, which is the functional region of the protein. These interactions, therefore, gave insight to the action of the drug compounds against the mutant protein.

**Figure 3.**
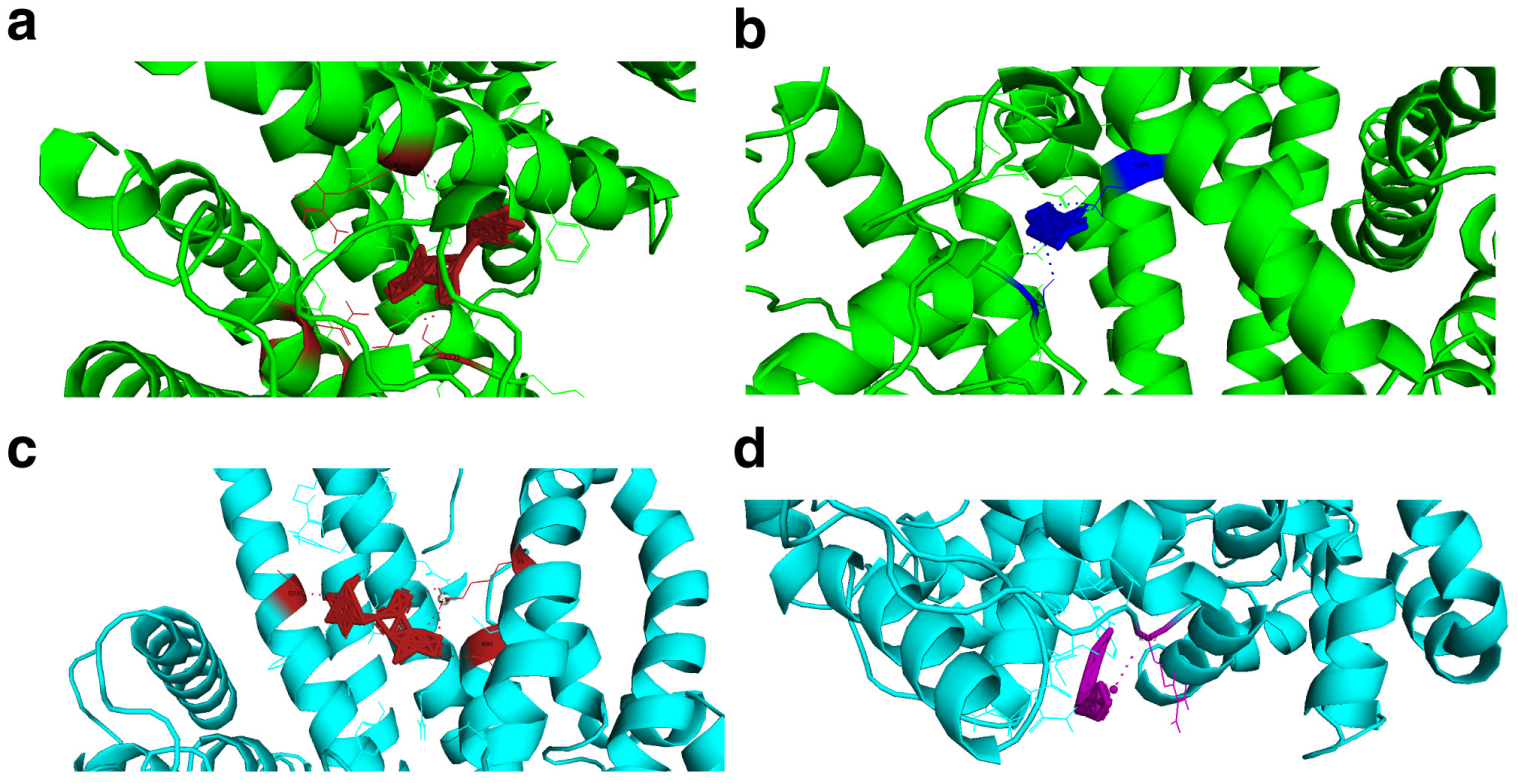
Visualization of protein-ligand interactions for both PHISTb/RLP1 reference and mutant proteins. ( **a** and **b**) visualizations of alpha carrageenan (firebrick) and fucoidan (blue) drug compounds interacting with PHISTb/RLP1 reference protein structure (green) at specific binding sites. The interacting residues and polar contacts are shown in firebrick and blue PyMOL colours respectively. ( **c** and **d**) are visualizations of alpha carrageenan (firebrick) and ghatti sulfate (purple) compounds interacting with PHISTb/RLP1 mutant protein structure binding pockets. The interacting residues and polar contacts are shown in firebrick and purple PyMOL colours, respectively. These specific interactions reveal potential inhibitory activity of these compounds against PHISTb/RLP1 proteins.

When selecting the poses after docking experiments, we selected the first pose of the AutoDock Vina pdbqt output file. The docking energies of the screened reference and mutant targets were very low. The energies released therefore supported the drug likeness of these compounds. The resulting Root Mean Square Deviation (RMSD) was zero (first pose RMSD are always zero). There was a difference in docking energies of the drug compounds interacting with PHISTb/RLP1 reference and mutant proteins because of the difference in binding site clusters. The interactions with different residues caused by the shifting of binding sites due to mutations resulted in the slight difference in energy released during docking ( Figure 3, Table 7– Table 9)

**Table 7.** Specific residue interactions of the drug compounds and the PHISTb/RLP1 reference protein structure as visualized in PyMOL by showing polar contacts. The highlighted residues show optimized interactions with amino acids that were predicted by the binding site predicting tools. Alpha carrageenan, beta carrageenan, amylopectin sulfate, cyclodextrin sulfate and fucoidan compounds interacted specifically with amino acids predicted in binding sites of the reference protein structure. They showed potential ability to clock functional domains of PHISTb/RLP1 protein.

Compound	Interacting residues PHISTb/RLP1 Reference Protein
Alpha Carrageenan	S132, R315, N80
Beta Carrageenan	K376, N408, E402, E403
Dextrin sulfate	K376, E377, Q404, D406, E402
Amylopectin sulfate	S201, N197, S132, K84, K205
Phenoxyacetyl cellulose sulfate/zinc sulfate	S113, Y137, T29, K111
Ghatti sulfate	K375, G317
2,4-Diaminoanisole sulfate	N7, S12
Cyclodextrin sulfate	S132, A133, F134
Fucoidan	K205, S132, A133
3,6-Di-O-benzoyl-D-galactal	N406, K371

**Table 8.** Specific amino acids of the PHISTb/RLP1 mutant protein interacting with different drug compounds at different clusters of the active sites. The highlighted residues are specific amino acids predicted by the binding sites prediction tool. Alpha carrageenan, ghatti sulfate and 3,6-Di-O-benzoyl-D-galactal compounds interacted optimally with predicted amino acids in the mutant protein binding sites. The other compounds interacted with amino acids within the PHIST and RESA domains of the protein. These interactions revealed potential activity of these compounds as inhibitors of PHISTb/RLP1 protein from carrying out its functions.

Compound	Interacting residue PHISTb/RLP1 Mutant Protein
Alpha carrageenan	K35, N90, N214, Q237
Beta carrageenan	D392, E402, K347
Dextrin sulfate	R315, E95
Amylopectin sulfate	D356, E353
Phenoxyacetyl cellulose sulfate/Zinc sulfate	S113, K261, N69, N121
Ghatti sulfate	R152
2,4-Diaminoanisole sulfate	N121, K125, L71
Cyclodextrin sulfate	E95, K94
Fucoidan	R318, N215, K94
3,6-Di-O-benzoyl-D-galactal	K19, N18, D17

**Table 9.** Docking energies of each drug compound on interacting with PHISTb/RLP1 reference and mutant proteins. The energies released were all very low, supporting the activity of the sulfated polysaccharides as drug compounds against the exported proteins. The low binding energies show high binding affinities. Generally, the docking energies in PHISTb/RLP1 mutant protein was slightly higher that in reference protein. As seen in the interaction results, the compounds are interacting with different residues in the two proteins hence the difference in the released energy.

	PHISTb/RLP1 WILD TYPE		PHISTb/RLP1 MUTANT	
Compound	affinity (kcal/mol)	RMSD upper bound	affinity (kcal/mol)	RMSD upper bound
Alpha carrageenan	-9.4	0	-7.9	0
Beta carrageenan	-8	0	-7.2	0
Dextrin sulfate	-11.9	0	-10.3	0
Amylopectin sulfate	-6.2	0	-5.9	0
Phenoxyacetyl cellulose sulfate/zinc sulfate	-4.7	0	-5.1	0
Ghatti sulfate	-6.6	0	-6.1	0
2,4-Diaminoanisole sulfate	-4.4	0	-4.7	0
Cyclodextrin sulfate	-7	0	-6.2	0
Fucoidan	-7.6	0	-7.2	0
3,6-Di-O-benzoyl-D- galactal	-9.4	0	-8.4	0

## Discussion

### Point mutations, protein structure analysis and binding sites

In *P. falciparum,* SNPs have been reported to cluster in subtelomeric regions of the chromosomes. A study comparing synteny of exported proteins in *P. vivax* and *P. falciparum* reported a large number of SNPs in chromosome 2 and 10 subtelomeric regions ( Sargeant *et al*., 2006). The subtelomeric regions of the *P. falciparum* genome have been reported to be highly variable. The genes found in these regions have shown sequence diversity within and across different *P. falciparum* isolates. The drug target PHISTb/RLP1 is located in chromosome 2. Our genetic diversity analysis of this protein in Kenyan isolates depicted many SNPs, with many of the polymorphisms having been identified before. Novel SNPs were reported with high frequency across our sample size. Novel point mutations were identified in our sequences across different groups of amino acids. These included I129T, T142V, Y147D, E154Q, S156H, T167I, S208L, M211T, L219H, D387N, D390N, and E403K. These substitutions are hypothesized to affect the function of the protein and we considered them to analyze their effect on the structure of the protein, as well as interactions with drug compounds.

The homology modelling of full length PHISTb/RLP1 protein revealed that the 3D structure of this drug target is characterized by alpha helices. This was confirmed by the solved PHIST domain crystal structure PDB ID 4JLE. Upon comparing the reference and mutant structures, there was a difference in the folding of the alpha helices. This difference was clearly depicted by the difference in clustering of the binding sites. This can be accounted for by the point mutations, which changed the conformation of the protein structure. The tool used to model the two proteins selected one different template for threading, which explains the difference in the overall writing of the pdb file for the two structures. The PHIST domain spans amino acids residues 1 to 167 of the protein sequence. Both structures had a cluster of functional sites towards the C-terminal. This emphasizes crucial function of this part of the protein. Other clusters were distributed in the middle and towards the N-terminal of the structures. The PHISTb/RLP1 is a multi-domain protein, it contains the PRESAN domain (Plasmodium-RESA N-terminal) and the RESA domain, which is a fusion of other domains, namely the DnaJ domain and the DnaJ-associated X domain Pfam accession number PF0987. The distribution of these domains across the protein sequence explains the clustering of the binding sites in the protein structure of the PHISTb/RLP1 protein.

### Sulfated polysaccharides drug compounds

Sulfated polysaccharides are a wide group of biochemical molecules with therapeutic properties including anti-thrombotic, anti-viral and anti-plasmodial activities ( Mourão, 2015). We narrowed down our search to sulfated polysaccharides with potential anti-malarial properties ( Boyle *et al*., 2017). The database search yielded ten compounds that are curated in PubChem compounds: Beta carrageenan, alpha carrageenan, dextrin sulfate, amylopectin sulfate, zinc sulfate, ghatti sulfate, 2,4-Diaminoanisole sulfate, cyclodextrin sulfate, Fucoidan,3-Aminophenylboronic acid and 3,6-Di-O-benzoyl-D-galactal. The compounds were tested for activity against exported protein PHISTb/RLP1 protein except for 3-Aminophenylboronic acid. Despite dextrin sulfate deviating from Lipinski’s Rule of Five, it was still tested. Dextrin sulfate has been supported by previous research to contain antimalarial activity ( Boyle *et al*., 2017). The drug activity of this compound supports its investigation as an antimalarial with further chemical modifications to suit rules of a drug compound. Carrageenan compounds antimalarial activity had previously been reported, however, the compound had to be further modified to reduce its toxicity ( Adams *et al*., 2005; Recuenco *et al*., 2014). All these compounds interacted with the PHISTb/RLP1 at different affinities and this inferred their inhibitory activity against the PHIST family of proteins.

### Interactions of sulfated polysaccharides with PHISTb/RLP1

The identified drug compounds interacted with the exported protein PHISTb/RLP1. Alpha carrageenan compound interacted with both the reference and the mutant proteins. The compound shows potential inhibitory activity against exported proteins. Amylopectin sulfate, cyclodextrin sulfate, ghatti sulfate, Fucoidan and 3,6-Di-O-benzoyl-D-galactal compounds have specific interactions with the protein. The interactions of the drug compounds with specific amino acids found in binding site clusters depicted that these compounds have the potential to block the protein domains used to invade red blood cells. 2,4-Diaminoanisole sulfate and zinc sulfate showed weak interactions with the proteins. The interactions with the mutant protein are generally weaker. The mutations found in the PHISTb/RLP1 changed the tertiary folding of the protein thus interfering with the active sites. The identified mutations I129T, T142V, Y147D, E154Q, S156H and T167I are within the PHIST domain (PDB ID: 4JLE) ( Oberli *et al*., 2014). I129T, T142V and T167I represent polar to non-polar and non-polar to polar substitutions. Y147D shows substitution of a polar amino acid to negatively charged aspartic acid residue. E154Q is a substitution from negatively charged to polar uncharged glutamine and S156H is a substitution from polar uncharged group to a positively charged amino acid. These changes affected the protein structure as well as enhancing the protein function. The binding affinity was different among the amino acids found in different structures and was shown by the difference in docking energies. The interactions of these compounds with exported proteins support the wide activity of sulfated polysaccharides against the *P. falciparum* parasite.

*P. falciparum* parasite uses the mechanism of exporting proteins to the host erythrocyte to enhance its virulence. These changes induced by the exported proteins include changing the physical properties of the cell and giving the cell adhesive properties. These changes enhance the pathogenesis of malaria in humans ( Boddey *et al*., 2016). Among the exported proteins, the PHIST family, which contains 89 proteins, has been identified to play a role in making the remodeled host cell more cytoadherent ( Warncke *et al*., 2016). The key target we have studied in this research, PHISTb/RLP1 (Pf3D7_0201600) plays a key role in remodeling of the host erythrocyte to enhance malaria virulence through cytoadherence mechanism ( Warncke *et al*., 2016). The PRESAN domain present in this protein contains the *Plasmodium* protein export element (PEXEL) motif that enables the export mechanism of the protein ( Boddey *et al*., 2016; Moreira *et al*., 2016). The interactions of the screened drug compounds with amino acids found in the functional domains of this protein reveal novel chemical inhibitors targeting exported proteins. The compounds have the ability to inhibit the PRESAN and PHIST domains from carrying out the export functions of the proteins. In *P. falciparum*, the PHIST family of proteins are expressed in the early and late ring stages, as well as trophozoites. Using PHISTb/RLP1 as a representative of this protein family, the interactions of the protein with sulfated polysaccharides infers that these compounds can deactivate exported proteins. The PHISTb proteins have been linked with controlling the *P. falciparum* Erythrocyte Membrane Protein ( *Pf*EMP1) major virulent factor of the parasite ( Tarr *et al*., 2014). The interactions of the screened drug molecules with the mutant protein of PHISTb/RLP1 show that even though the mutations change the protein folding, the functional domains are still being blocked by the chemicals. The screened sulfated polysaccharides adhere to the Lipinski Rule of Five. Their toxicity levels are very low and can be investigated further in *in vitro* analysis and clinical trials.

## Conclusion

The interactions of specific sulfated polysaccharide compounds with PHISTb/RLP1 protein are the first findings showing compounds that can act against exported proteins of the *Plasmodium* parasite. These findings support further drug discovery downstream processes with these compounds as lead compounds for developing the next class of antimalarial agents. Crystal structures solving PHISTb/RLP1 and other exported proteins is recommended to enable more insight into the implications of structural variants on the protein structure and functions.

## Data availability

Open Science Framework: Novel Characterization of Sulfated Polysaccharides Activity against Virulent Plasmodium falciparum PHIST Proteins, https://doi.org/10.17605/OSF.IO/YFQAZ ( Mutisya, 2020).

This project contains the following underlying data:

- Sequenced reads of PHISTb/RLP1 acquired through Sanger sequencing
- Multiple Sequence Alignment files
- Protein .pdb files (of both PHISTb/RLP1 reference and mutant protein structures)
- Sulfated polysaccharides .sdf and .pdbqt files.

Data are available under the terms of the Creative Commons Zero "No rights reserved" data waiver (CC0 1.0 Public domain dedication).
