# Supplementary material for: Characterization of sulfated polysaccharide activity against virulent Plasmodium falciparum PHISTb/RLP1 protein
Source: F1000Res. 2022 Apr 29;9:1268. Originally published 2020 Oct 23. [Version 2] doi: 10.12688/f1000research.26756.2 (PMC9096147; doi:10.12688/f1000research.26756.2)
Supplement: Peer review history

# Peer review: Reviewer response for version 2

**DOI:** 10.5256/f1000research.122945.r136370

**Published:** 2022-05-11

**Authors:** Eusébio Macete

**Affiliations:** 1 Centro de Investigação em Saúde de Manhiça (CISM), Maputo, Mozambique

Thanks for the responses, from my point of view the article can be indexed.

Is the work clearly and accurately presented and does it cite the current literature?

Yes

If applicable, is the statistical analysis and its interpretation appropriate?

I cannot comment. A qualified statistician is required.

Are all the source data underlying the results available to ensure full reproducibility?

Yes

Is the study design appropriate and is the work technically sound?

Yes

Are the conclusions drawn adequately supported by the results?

Yes

Are sufficient details of methods and analysis provided to allow replication by others?

Yes

Reviewer Expertise:

NA

I confirm that I have read this submission and believe that I have an appropriate level of expertise to confirm that it is of an acceptable scientific standard.

# Peer review: Reviewer response for version 2

**DOI:** 10.5256/f1000research.122945.r136371

**Published:** 2022-05-03

**Authors:** Abraham Madariaga

**Affiliations:** 1 Institute of Chemistry, National Autonomous University of Mexico, Mexico City, Mexico

I agree with the new version of the manuscript, the authors have addressed most of the reviewers' comments. I have no further comments.

Is the work clearly and accurately presented and does it cite the current literature?

Yes

If applicable, is the statistical analysis and its interpretation appropriate?

Not applicable

Are all the source data underlying the results available to ensure full reproducibility?

Partly

Is the study design appropriate and is the work technically sound?

Partly

Are the conclusions drawn adequately supported by the results?

No

Are sufficient details of methods and analysis provided to allow replication by others?

No

Reviewer Expertise:

Molecular modeling including docking and molecular dynamics simulations; QSAR studies, homology modeling

I confirm that I have read this submission and believe that I have an appropriate level of expertise to confirm that it is of an acceptable scientific standard.

# Peer review: Reviewer response for version 1

**DOI:** 10.5256/f1000research.29544.r121686

**Published:** 2022-03-01

**Authors:** Abraham Madariaga

**Affiliations:** 1 Institute of Chemistry, National Autonomous University of Mexico, Mexico City, Mexico

The manuscript describes the modeling of a protein and its mutant from Plasmodium falciparum, and the assessment of some ligands with these models by molecular docking analysis. The mutant was built based on experimental data from several samples. Both protein models were validated using online tools. Although the authors used several online tools for assessing the structural performance of the proteins, the mutant model is lacking confidence in its tridimensional structure. Furthermore, the docking protocol is not fully described. The results are too preliminary to suggest a potential activity of the selected ligands against PHISTb/RLP1 proteins. I would recommend major revision prior to indexing.

 The modeled mutant protein resulted with a very different tridimensional configuration to the reference protein. For better prediction and assessment of a homology model, it is suggested to perform a molecular dynamics simulation of at least 50 ns in order to let the structure correctly minimize. Otherwise, the predicted protein may have a non-biological relevance.

 It is not clear why the authors select the fourth model of each structure (reference and mutant) for energy minimization. Does LOMETs give a score? Furthermore, the TM score is significantly high for both structures, meaning a “perfect match” between the initial model and the generated model. It is surprising that the mutant model gets a TM as similar as the reference model, so the mutant has no apparent structural changes despite having a dozen different amino acids. Nevertheless we can see in Fig2a-b big differences among those structures. How do you explain this?

 Rule of five is not synonymous with toxicity. Rule of five gives an empirical approximation of the pharmacokinetics of a molecule, in terms of absorption. Ro5 is a simple approach intended to select molecules with druggability properties. To examine toxicity of molecules, many other methods can be used (Ames mutagenicity prediction, ADME-Tox predictions, LD50, etc) in different online platforms.

 Docking protocol. Were the ligands used in their salt forms? For better results, the ligands should undergo a “washing” procedure that includes removing contra ions, adjusting charges, and geometric minimizing process. It is not described in the methodology. 

 Regarding the gridbox, what size does it have? Depending on the size, the ligands (in this case the ligands are large molecules) are allowed to freely rotate or not. This may bias the docking results.

 On the other hand, if a positive control is not used as a part of the docking experiments, it is difficult to argue that a molecule will have a potent activity, such in the case of the manuscript. It is preferable to suggest only a possible activity. 

 For completeness of the discussion, a figure depicting the ligand-protein interaction of the ligands with better affinity would be great.

 Minor

 Page 6, Section “Preparation of proteins and ligands, …”, line 8: Please change “autodock tolls” for “autodock tools”.

Is the work clearly and accurately presented and does it cite the current literature?

Yes

If applicable, is the statistical analysis and its interpretation appropriate?

Not applicable

Are all the source data underlying the results available to ensure full reproducibility?

Partly

Is the study design appropriate and is the work technically sound?

Partly

Are the conclusions drawn adequately supported by the results?

No

Are sufficient details of methods and analysis provided to allow replication by others?

No

Reviewer Expertise:

Molecular modeling including docking and molecular dynamics simulations; QSAR studies, homology modeling

I confirm that I have read this submission and believe that I have an appropriate level of expertise to confirm that it is of an acceptable scientific standard, however I have significant reservations, as outlined above.

# Response

**Published:** 2022-04-18

**Authors:** Jennifer Mutisya

**Affiliations:** Jomo Kenyatta University of Agriculture and Technology, Kenya

Comment: The manuscript describes the modeling of a protein and its mutant from Plasmodium falciparum, and the assessment of some ligands with these models by molecular docking analysis. The mutant was built based on experimental data from several samples. Both protein models were validated using online tools. Although the authors used several online tools for assessing the structural performance of the proteins, the mutant model is lacking confidence in its tridimensional structure. Furthermore, the docking protocol is not fully described. The results are too preliminary to suggest a potential activity of the selected ligands against PHISTb/RLP1 proteins. I would recommend major revision prior to indexing.

**Response: All the tools used in modeling and assessment of the protein structures are open source tools with algorithms that are assessed and published. Furthermore, they have been used and cited in other scientific research prior to this work hence able to provide significant scientific data as documented in the provided links and cited publications for each tool used. The docking protocol details are included; that is preparation of ligand files in MGL tools, grid box parameters and python script used.**

 Comment: The modeled mutant protein resulted with a very different tridimensional configuration to the reference protein. For better prediction and assessment of a homology model, it is suggested to perform a molecular dynamics simulation of at least 50 ns in order to let the structure correctly minimize. Otherwise, the predicted protein may have a non-biological relevance.

**Response: Energy minimization carried out using ModRefiner an algorithm for atomic-level, high-resolution protein structure refinement, which can start from either C-alpha trace. The models underwent energy minimization using this tool prior to docking experiments. Molecular dynamics simulation of at least 50ns were not possible to carry out due to minimal expertise in a tool that can do that. However, after energy minimization using ModRefiner the models quality improved and were fit for docking simulations.**

 Comment: It is not clear why the authors select the fourth model of each structure (reference and mutant) for energy minimization. Does LOMETs give a score? Furthermore, the TM score is significantly high for both structures, meaning a “perfect match” between the initial model and the generated model. It is surprising that the mutant model gets a TM as similar as the reference model, so the mutant has no apparent structural changes despite having a dozen different amino acids. Nevertheless, we can see in Fig2a-b big differences among those structures. How do you explain this?

**Response: The fourth model was chosen after screening the Ramachandran score for the five suggested models hence the fourth model had the highest scores. Lomets does not provide scores for models since they are racked from number 1 to 5, but for the template selected. Structures match because the middle template selected by the algorithm was similar in both reference and mutant modelling jobs. The PHIST domain is the only present crystal structure for this protein family in PDB. This is why the TM score matches closely. The difference between the two structures can be seen in the alpha-helix folding which is significantly distinct and postulated to be an effect of the SNPs identified.**

 Comment: Rule of five is not synonymous with toxicity. Rule of five gives an empirical approximation of the pharmacokinetics of a molecule, in terms of absorption. Ro5 is a simple approach intended to select molecules with draggability properties. To examine the toxicity of molecules, many other methods can be used (Ames mutagenicity prediction, ADME-Tox predictions, LD50, etc) in different online platforms.

**Response: ADMET analysis data was included using SwissADME tool as suggested.**

 Comment: Docking protocol. Were the ligands used in their salt forms? For better results, the ligands should undergo a “washing” procedure that includes removing contra ions, adjusting charges, and geometric minimizing process. It is not described in the methodology. 

**Response: The ligand files were converted using the Open Babel tool and prepared using MGL tools as described in the text. This editing enabled the removal of contradicting s ions prior to docking.**

 Comment: Regarding the grid box, what size does it have? Depending on the size, the ligands (in this case the ligands are large molecules) are allowed to freely rotate or not. This may bias the docking results.

**Response: grid box dimensions were added in the main text.**

 Comment: On the other hand, if positive control is not used as a part of the docking experiments, it is difficult to argue that a molecule will have a potent activity, such in the case of the manuscript. It is preferable to suggest only a possible activity. 

**Response: Included low molecular weight heparin as the positive control compound since it is a well-researched sulfated polysaccharide with anti-malarial properties.**

 Comment: For completeness of the discussion, a figure depicting the ligand-protein interaction of the ligands with better affinity would be great.

**Response: Included a figure in the discussion**

# Peer review: Reviewer response for version 1

**DOI:** 10.5256/f1000research.29544.r100719

**Published:** 2022-01-31

**Authors:** Eusébio Macete

**Affiliations:** 1 Centro de Investigação em Saúde de Manhiça (CISM), Maputo, Mozambique

My first comment is regarding the selection of the sites, the authors did not mention how the sites were selected.

 Were the samples collected for this specific study? Or is this study a secondary analysis? If yes, what was the primary study?

 Second, in the article the authors mentioned that the samples were tested for plasmodium falciparum, so, how were patients that had positive results treated? If that was the case?

 In the method paragraph, the study design is not clear. How were the samples selected etc.

Is the work clearly and accurately presented and does it cite the current literature?

Yes

If applicable, is the statistical analysis and its interpretation appropriate?

I cannot comment. A qualified statistician is required.

Are all the source data underlying the results available to ensure full reproducibility?

Yes

Is the study design appropriate and is the work technically sound?

Yes

Are the conclusions drawn adequately supported by the results?

Yes

Are sufficient details of methods and analysis provided to allow replication by others?

Yes

Reviewer Expertise:

I am specialist in public health working mostly in malaria drug and vaccines.

I confirm that I have read this submission and believe that I have an appropriate level of expertise to confirm that it is of an acceptable scientific standard, however I have significant reservations, as outlined above.

# Response

**Published:** 2022-02-07

**Authors:** Jennifer Mutisya

**Affiliations:** Jomo Kenyatta University of Agriculture and Technology, Kenya

Concerning the comments on sample sites selection and collection:

 The samples used in this study were not collected specifically for this secondary study but were used on basis of availability. The samples were collected for a bigger project that studies drug resistance patterns from the identified regions. The sampling for this study was hence non-randomly conducted from sample inventory of archived whole blood samples. This study was a sub-study in the Malaria Drug Resistance Laboratory and not the primary study of the institution. The sample sites were selected according to different Malaria endemic zones in Kenya and were guided by the proposal of the main study which studies epidemiology patterns of the disease in Kenya.

 The patients who tested positive were treated with recommended Artemesinin combined therapies at day zero of testing and follow up samples were collected after 7, 14 and 28 days. These were done according to the objectives of a different study. The current study did not need the treatment data.
